# COVID-19 Vaccine Readiness Among Acute-Care Registered Nurses in New Jersey: Results of a Statewide Survey

**DOI:** 10.1017/ash.2021.106

**Published:** 2021-07-29

**Authors:** Monika Pogorzelska-Maziarz, Mary Lou Manning, Angela Gerolamo, Mary Johansen, Irina Grafova, Suzie Crincoli, Pamela de Cordova

## Abstract

**Background:** The coronavirus disease 2019 (COVID-19) vaccine is an important intervention to control the COVID-19 pandemic. As the most trusted profession integral to providing care to patients across all care settings, nurses play a critical role in educating patients regarding the SARS-CoV-2 vaccine. However, little is known about the readiness of registered nurses (RNs) to receive the vaccine. **Methods:** In October 2020, prior to FDA approval of vaccines, we conducted a cross-sectional electronic survey of all active registered nurses in the state of New Jersey. The eligibility criteria included providing direct patient care in a New Jersey hospital in an emergency or an adult inpatient unit during the emergence of COVID-19 (March 2020). **Results:** In total, 3,027 RNs completed the survey (15% response rate). When asked whether they plan to get vaccinated, 27% of RNs responded yes, 30% responded no, and 43% were undecided. Among those RNs who reported that they were planning to get vaccinated, their main reasons for their willingness to receive the vaccine included (1) wanting to protect themselves and their families (95%), (2) wanting to protect the community at large (76%), wanting to protect their patients (75%), the belief that life won’t get back to normal until most people are vaccinated (72%), and the belief that getting vaccinated is the best way to avoid getting seriously ill from COVID-19 (67%). The main reasons reported for not planning to or being undecided about getting vaccinated included the belief that the vaccine will likely be developed too quickly to be safe (81%) and concern about the side effects from the vaccine (74%). RNs also reported being in a low-risk group for becoming seriously ill (12%) and having had COVID-19 (8%) as reasons for planning not to get vaccinated. In open-ended responses, participants also discussed several additional issues driving vaccine hesitancy: their lack of trust in the political process, planning to become pregnant or currently pregnant or breastfeeding, questions about effectiveness of the vaccine and long-term side effects, and the need for more information before making a decision. **Conclusions:** This cross-sectional study of all acute-care RNs in the State of New Jersey was conducted prior to the FDA approval of COVID-19 vaccines. The results outline factors driving vaccine hesitancy among RNs. Although vaccine efficacy data and approval by the FDA may have alleviated some of these fears, immunization programs for healthcare workers and the public should focus on dispelling myths about vaccine development and side effects.

**Funding:** No

**Disclosures:** None

